# Effect of Post-Extraction Ultrasonication on Compositional Features and Antioxidant Activities of Enzymatic/Alkaline Extracts of *Palmaria palmata*

**DOI:** 10.3390/md22040179

**Published:** 2024-04-17

**Authors:** Sakhi Ghelichi, Ann-Dorit Moltke Sørensen, Mona Hajfathalian, Charlotte Jacobsen

**Affiliations:** National Food Institute, Technical University of Denmark, 2800 Kongens Lyngby, Denmark; saghel@food.dtu.dk (S.G.); adms@food.dtu.dk (A.-D.M.S.); monhaj@food.dtu.dk (M.H.)

**Keywords:** *Palmaria palmata*, enzymatic/alkaline extraction, protein hydrolysate, post-extraction ultrasonication, antioxidant activity, phenolic compounds

## Abstract

*Palmaria palmata* is a viable source of nutrients with bioactive properties. The present study determined the potential role of post-extraction ultrasonication on some compositional features and antioxidant properties of enzymatic/alkaline extracts of *P. palmata* (EAEP). No significant difference was detected in terms of protein content and recovery, as well as the amino acid composition of the extracts. The nitrogen-to-protein conversion factor of 5 was found to be too high for the seaweed and EAEP. The extracts sonicated by bath for 10 min and not sonicated showed the highest and lowest total phenolic contents (*p* < 0.05), respectively. The highest radical scavenging and lowest metal-chelating activities were observed for the non-sonicated sample, as evidenced by IC_50_ values. The extract sonicated by bath for 10 min showed the most favorable in vitro antioxidant properties since its radical scavenging was not significantly different from that of the not-sonicated sample (*p* > 0.05). In contrast, its metal-chelating activity was significantly higher (*p* < 0.05). To conclude, post-extraction ultrasonication by an ultrasonic bath for 10 min is recommended to increase phenolic content and improve the antioxidant properties of EAEP.

## 1. Introduction

The health-promoting effects of bioactive peptides and amino acids have made them stand out as natural and harmless candidates for developing functional foods and nutraceuticals [1,2,3]. Their significance becomes apparent when considering their biological roles in the human body and nutritional value due to their content of essential amino acids [4]. Bioactive peptides and amino acids from marine sources exert a broad spectrum of biological effects on the cardiovascular, immune, nervous, and gastrointestinal systems [5]. However, originating mostly from fish and mollusks, most marine-derived proteins and peptides are not accepted by vegan and vegetarian communities. Therefore, using marine sources of bioactive nutrients that are also compatible with the standards of these communities could be a research priority.

Seaweed has traditionally been considered a food source consumed either directly or as a food supplement with medicinal effects. It has been documented that local inhabitants near the shores of many parts of the world have harvested seaweed and used it as a home remedy and natural marine drug for treating different diseases [6]. In recent years, advancements in the study of natural products derived from marine algae have revealed their potential as valuable sources of bioactive compounds with potential medicinal applications [7,8]. The red seaweed dulse (*Palmaria palmata*) is regarded for its appealing taste with potential in culinary applications, both as a stand-alone food and as an ingredient. This seaweed species is abundant in cold Atlantic waters [9] and can be harvested from the wild or cultivated in sea and land-based pools [10]. *P. palmata* has been introduced as a viable alternative source of nutrients such as proteins, peptides, and amino acids [11] with possible medicinal effects [12,13]. For instance, peptides obtained by protein hydrolysis could be used in the formulation of pharmaceuticals and nutraceuticals as an antidiabetic agent [13]. A recent study even discovered the role of nutrients in seaweed to make it a promising prebiotic potentially used as a neuroprotective agent in multiple sclerosis patients [14].

Despite the bioactivity and health benefits of proteins and peptides from the seaweed, their extraction from the species is not a straightforward process, mainly due to its rigid cell wall with its complex backbone-comprising polysaccharides, proteins, and polyphenols [9], which poses a challenge in reaching intracellular nutrients. One remedy to overcome this barrier is the application of polysaccharidase to disintegrate the cell wall, which has been reported to yield favorable protein recovery from the seaweed. For instance, it was shown that applying a polysaccharidase and a protease in the aqueous solution and sequentially performing alkaline extraction would facilitate the favorable recovery of protein from this seaweed [11]. Since proteins are hydrolyzed during enzymatic/alkaline extraction, the resulting extract should contain bioactive peptides of smaller sizes and even free amino acids. Peptides and amino acids in protein hydrolysates from other sources are shown to have antioxidant properties [15]. Likewise, peptides obtained from other seaweed species have been demonstrated to have antioxidative properties [16]. The seaweed extracts could also act as antioxidants via other nutrients such as phenolic compounds. Therefore, there are still controversies on whether the antioxidant properties of seaweed-derived extracts, either in terms of radical scavenging or metal chelation, originated from peptides and amino acids or polyphenols or even other nutrients such as polysaccharides [17]. Therefore, we bolstered our findings by presenting polyphenol data. These results emphasize that the observed effects within the extracts cannot be exclusively attributed to peptides and amino acids.

The application of enzymatic treatment involving a protease leads to protein hydrolysis, yielding a variety of peptides with distinct amino acid compositions. Our analysis of protein content, following the standard nitrogen-based approach, was complemented by examining amino acid profiles. These data allowed us to assess the nitrogen-to-protein conversion rate meticulously. Conventional studies have used a nitrogen-to-protein conversion factor of 6.25, but recent research suggests that this overestimates seaweed protein content and instead favors a factor of 5 [18]. Additionally, measuring protein content based on the sum of amino acids is a well-established method [19]. Our study further enhances accuracy by considering the water gained during amino acid hydrolysis.

In addition, all the studies analyzing the antioxidant effects of seaweed extracts applied the crude extract, whether as a liquid fraction or powdered form, without further treatment. However, we hypothesized that the crude EAEP could exert more favorable effects with additional treatment.

Ultrasonication is reputable as a green treatment technique with many applications. It has been widely employed during alkaline protein extraction from plants [20]. Regarding seaweeds, ultrasonication has been directly applied for extracting bioactive compounds [21] or used as a pretreatment to disrupt rigid cell walls [22]. However, despite these applications, there is still room for further exploration. To date, no reports have explored the impact of ultrasonication on seaweed (or even plant) extracts after extraction when the biomass is practically removed. The theoretical framework in this study regarding the effect of ultrasonicating on the extracts after extraction is as follows: (i) the stabilization of extracted bioactive compounds by preserving them from degradation; (ii) the smaller size of bioactive compounds that improve their solubility, bioavailability, and bioactivity; (iii) the disruption of aggregates and crystalline structure that would increase their availability in the treated extracts; (iv) the degassing effect of ultrasonication that would remove dissolved gases such as oxygen from the extracts contributing to higher stability in terms of the oxidation of bioactive compounds; and (v) the further release of bioactive compounds from unknown agglomerated structures such as cell remnants. Additionally, there are generally two distinct methods for ultrasonication, i.e., bath and probe, which are known to exert ultrasonic energy in different ways and, thus, yield different results [23]. Therefore, this study aimed to evaluate whether ultrasonication treatment of EAEP would influence compositional features and the antioxidative properties of the extract. The second aim was to compare two different ultrasonication methods: ultrasound bath versus ultrasound probe at two different treatment times.

## 2. Results

### 2.1. Protein Content, Degree of Hydrolysis, Protein Recovery, and Nitrogen-to-Protein Conversion Factor

The protein content of freeze-dried seaweed and EAEP (% dry matter), degree of hydrolysis (DH), and protein recovered in hydrolysates and maintained in solid residues collected after enzymatic hydrolysis and alkaline extraction are presented in Table 1. Furthermore, the protein recovered in hydrolysates and solid residues was circa 91–94% and 3–5%, respectively. No significant difference was detected in terms of protein recovery in hydrolysates and solid residues (*p* > 0.05). The DH of the hydrolysates ranged between circa 25% and 33%; however, no significant difference was detected among the samples in terms of DH (*p* > 0.05). A substantial amount of protein in the raw material was recovered in the hydrolysates, as evidenced by >91% protein recovery in all the samples.

Table 2 compares the protein contents of dried seaweed and EAEP, considering three conversion factors (6.25, 5, and 4), and accurate protein content and conversion factors, considering the estimated total amino acids. The results of this study in terms of amino acid contents (See Section 2.3) revealed that the conversion factor of 4.6–4.7 could be a viable choice for the estimation of the protein content of EAEP.

### 2.2. Total Phenolic Compounds

**Figure 1:**
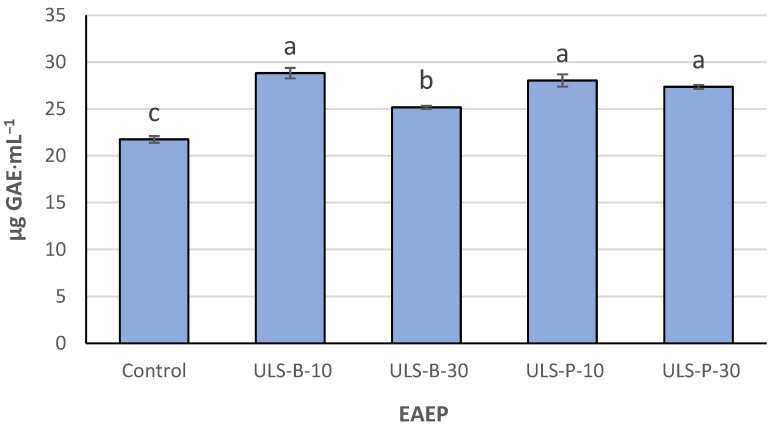
Total phenolic compounds (TPC) of EAEP. Data were expressed as mean ± standard deviation (*n* = 2). Control: no ultrasonication; ULS-B-10: ultrasonication by bath for 10 min; ULS-B-30: ultrasonication by bath for 30 min; ULS-P-10: ultrasonication by probe for 10 min; ULS-P-30: ultrasonication by probe for 30 min. The letters ‘a’, ‘b’, and ‘c’ denote significant differences among the treatments (*p* < 0.05).

Total phenolic compounds (TPC) content underwent considerable change after the ultrasonication of EAEP (Figure 1). TPC in the control sample (no ultrasonication) was significantly lower than that in ultrasonicated EAEP (*p* < 0.05). The highest TPC was found in ULS-B-10 (ultrasonic bath for 10 min), but it was not significantly different from values obtained in EAEP ultrasonicated using a probe for 10 and 30 min (*p* > 0.05). However, the TPC of ULS-B-30 (ultrasonic bath for 30 min) was significantly lower than that of other ultrasonicated extracts (*p* < 0.05) but still significantly higher than that of the control (*p* < 0.05), which further highlighted the substantial effect of ultrasonication on the content of the phenolic compounds of the extracts.

### 2.3. Amino Acid Composition

Table 3 summarizes the amino acid composition of freeze-dried seaweed and EAEP with or without following ultrasonication either by ultrasonic bath or ultrasound probe. The raw material and hydrolysate contained most amino acids except for tryptophan, cysteine, and hydroxyproline. Additionally, histidine was found in the freeze-dried seaweed but not the hydrolysates.

There was a tendency for the content of all amino acids to decrease with extended exposure of extracts to ultrasonic energy in the bath (*p* > 0.05). However, when comparing the hydrolysates obtained after 10 and 30 min of ultrasonication by the probe, there were fluctuations in the amino acid quantities (*p* > 0.05). Furthermore, the proportion of essential amino acids relative to non-essential amino acids rose from nearly 35% in the raw material to approximately 42% in the hydrolysates.

### 2.4. In Vitro Antioxidant Properties

#### 2.4.1. 1,1-Diphenyl-2-picrylhydrazyl (DPPH) Radical Scavenging Activity

**Figure 2:**
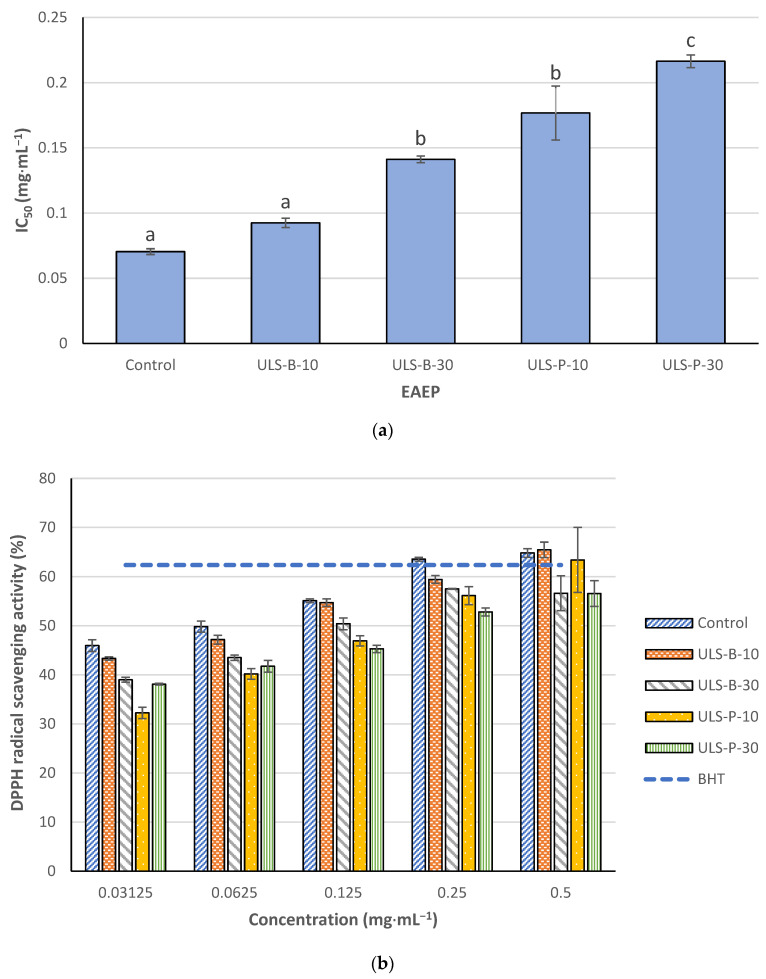
DPPH radical scavenging activity of EAEP: (**a**) IC_50_ values; (**b**) dose-dependent comparison with BHT (0.2 mg·mL^−1^) as a positive control. Data were expressed as mean ± standard deviation (*n* = 2). Control: no ultrasonication; ULS-B-10: ultrasonication by bath for 10 min; ULS-B-30: ultrasonication by bath for 30 min; ULS-P-10: ultrasonication by probe for 10 min; ULS-P-30: ultrasonication by probe for 30 min. The letters ‘a’, ‘b’, and ‘c’ denote significant differences among the treatments (*p* < 0.05).

Figure 2a depicts the IC_50_ values for DPPH radical scavenging activity of EAEP. The strongest DPPH radical scavenging activity was observed for the extracts obtained without ultrasonication or ultrasonicated for 10 min in the bath with IC_50_ values lower than 0.1 mg·mL^−1^ (*p* < 0.05). Continuing ultrasonication in the bath for 30 min resulted in a significant increase in IC_50_ value and, therefore, a substantial decrease in the DPPH radical scavenging activity of the extract (*p* < 0.05). Ultrasonication using the probe significantly decreased the DPPH radical scavenging activity of the extracts compared with the ones with no ultrasonication or ultrasonication in the bath for 10 min (*p* < 0.05). Moreover, the results revealed that the DPPH radical scavenging activity of the extract ultrasonicated for 30 min using a probe was significantly lower than that of other samples, as evidenced by significant differences in IC_50_ values of this extract with its counterparts (*p* < 0.05). DPPH radical scavenging activity of EAEP increased dose-dependently (Figure 2b). In this study, butylated hydroxytoluene (BHT) at 0.2 mg·mL^−1^ was used as a positive control. When applied at 0.25 mg·mL^−1^, only the control sample reached the same DPPH radical scavenging activity as the positive control. However, at a higher concentration of 0.5 mg·mL^−1^, in addition to the control, the extracts ultrasonicated (whether by bath or probe) for 10 min also reached almost the same DPPH scavenging activity of BHT (0.2 mg·mL^−1^).

#### 2.4.2. Fe^2+^ Chelating Activity

**Figure 3:**
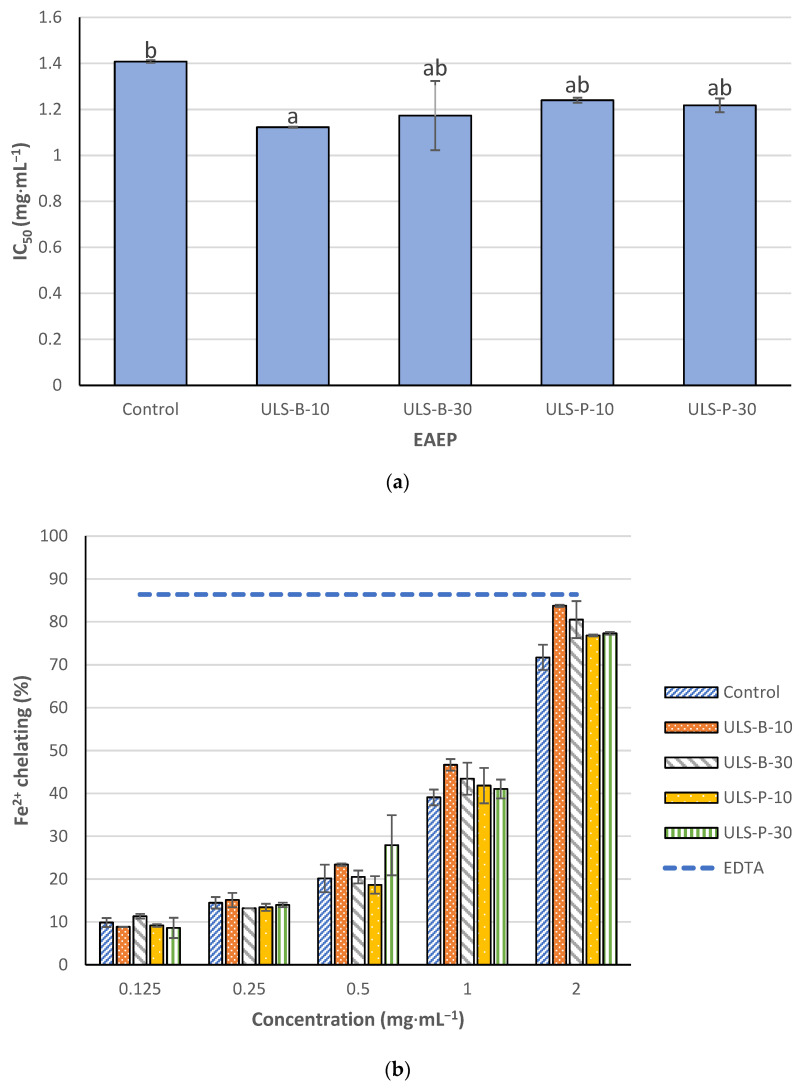
Fe^2+^ chelating activity of EAEP: (**a**) IC_50_ values; (**b**) dose-dependent comparison with 0.06 mM EDTA as a positive control. Data were expressed as mean ± standard deviation (*n* = 2). Control: no ultrasonication; ULS-B-10: ultrasonication by bath for 10 min; ULS-B-30: ultrasonication by bath for 30 min; ULS-P-10: ultrasonication by probe for 10 min; ULS-P-30: ultrasonication by probe for 30 min. The letters ‘a’ and ‘b’ denote significant differences among the treatments (*p* < 0.05).

The IC_50_ values of the extracts to chelate Fe^2+^ are illustrated in Figure 3a. In contrast to DPPH radical scavenging activity, the lowest Fe^2+^ chelating activity was detected in the control sample, which had no significant difference with samples ultrasonicated for 30 min in the bath and both samples ultrasonicated using the probe (*p* > 0.05). Conversely, a significant difference was witnessed in the iron chelating activity of the samples without ultrasonication and ultrasonicated for 10 min in the bath (*p* < 0.05). As expected, the iron chelating activity of the extracts showed a direct correlation with the dose applied (Figure 3b). In the present study, 0.06 mM ethylene diamine tetra acetic acid (EDTA) was a positive control and showed high Fe^2+^ chelating activity. None of the extracts, even when tested at a concentration of 2 mg·mL^−1^, could reach the threshold defined by the iron chelating activity of the positive control.

#### 2.4.3. In Vitro Antioxidant Activity of EAEP versus Other Types of Extracts

A comparative analysis of IC_50_ values, assessing the in vitro antioxidant activities of EAEP (in this study) alongside extracts from *P. palmata* obtained using different solvents (ethanol, water, chloroform, ethyl acetate, and methanol), as reported in other studies, is illustrated in Table 4. Notably, the IC_50_ values for DPPH radical scavenging in our study were substantially lower across all extracts than those observed in ethanol and water extracts [17] and chloroform, ethyl acetate, and methanol extracts [24]. However, for Fe^2+^ chelating activity, the IC_50_ values in ethanol and chloroform extracts remained lower than those of EAEP.

## 3. Discussion

### 3.1. Protein Content, Degree of Hydrolysis, Protein Recovery, and Nitrogen-to-Protein Conversion Factor

The dried seaweed’s protein content corresponded with the harvest season of the *P. palmata*. The seaweed studied in the present study was obtained from a batch harvested between late spring and early autumn, when the protein content was expected to be the lowest due to limited water nutrients during these months and the destructive effect of sunlight on proteins [25].

Unrealistic reports of protein content in *P. palmata* (e.g., 35%) can be found in the literature, which should be taken with care due to the possible risk of overestimation when applying the nitrogen-to-protein conversion factor of 6.25 (compared with 5 considered in this study) [26]. This discrepancy in the protein content of *P. palmata* could be due to non-protein nitrogenous compounds such as ammonium salts, amines, and nitrates [27]. It has been stated that direct amino acid analysis resonates as the most accurate method of protein quantification, especially in emerging alternative protein sources such as seaweed. It provides a ground on which the accuracy of other methods, such as Kjeldal and DUMAS, may be biased [28]. Nevertheless, it is noteworthy that the process taken in the present study to measure amino acid content does not determine all the amino acids. Furthermore, the gained water during the protein hydrolysis stage should also be considered to adopt total amino acid content as a measure of protein content. As can be seen in Table 2, considerably lower nitrogen-to-protein conversion factors were obtained by accounting for the water gained during protein hydrolysis to measure protein content based on total amino acids in the biomass and hydrolysates. The conversion factor was 4.54 for the dried seaweed and 3.91–4.20 for the hydrolysates. Therefore, one should think twice before calculating the protein content and, consequently, protein recovery in seaweed (or, at least, *P. palmata*) extracts via the nitrogen-to-protein conversion factor of 5, because the results of the present study revealed that the conversion factor of 5 might overestimate the actual protein content in seaweed extracts and even in raw biomass. It is worth noting that the conversion factors calculated here are for the hydrolysates obtained after sequential enzymatic and alkaline treatments. In contrast, another study determining the conversion factors of enzymatically obtained (yet, by using different enzymes) hydrolysates (i.e., without pursuing alkaline extraction) reported much lower conversion factors for liquid extracts ranging from 2.5 to 3.6 [27]. It needs to be evaluated whether these differences are due to the additional alkaline extraction stage in this study or the different choices of enzymes in the present study (Celluclast + Alcalase) versus the above-mentioned research (Xylanase, Xylanase + Umamizyme and Umamizyme). It is noteworthy that the conversion factor for freeze-dried *P. palmata* in the above study (i.e., 4.7) was quite comparable to the ones calculated in the current research (i.e., 4.8 or 4.54, as explained above), which downplays the risk of discrepancies caused by external factors such as accuracy of measurements and/or human errors.

DH of the hydrolysates ranged between circa 25% and 33%; however, no significant difference was detected among the samples in terms of DH (*p* > 0.05). Data on the degree of hydrolysis for seaweed is sparse, making it challenging to compare based on the enzymes adopted, species of seaweed, and hydrolysis conditions such as time, temperature, and pH. However, Alcalase has been reported to yield favorable DH when used to hydrolyze protein from different sources [29]. Research is required to discover the effect of using different enzymes simultaneously or sequentially on the degree of hydrolysis. Furthermore, since a polysaccharidase was used here to disintegrate the cell wall of the seaweed to facilitate the protein hydrolysis by the protease, possible synergism and/or antagonism between the protease and polysaccharidase in terms of their effect on the degree of hydrolysis merit consideration.

A substantial amount of protein in the raw material was recovered in the hydrolysates, as evidenced by >91% protein recovery in all the samples. In a previous study from our lab [11], where the pH was shifted from approximately 13 to 3 after combined enzymatic/alkaline extraction, the protein recovery in the liquid fractions (called hydrolysates in the present study) ranged between circa 56% and 70% when Alcalase was used in combination with other enzymes such as Celluclast. The results indicate that the pH adjustment of EAEP to a range between 8.5 and 9 contributed to the solubility of a significant quantity of resulting peptides and amino acids. The pH range of 8–9 was recommended as the most favorable range for protein solubility since proteins have zero charge at their isoelectric point and form zwitterion structures leading to protein aggregation and, thus, minimized solubility [30]. The substantial increase in the solubility of seaweed protein in enzymatic/alkaline extracts may be attributed to different factors. The high ionic strength resulting from mineral content may contribute to the elevated solubility of proteins at alkaline pH [31]. Additionally, the availability of hydrophobic and free sulfhydryl groups for interaction because of protein unfolding via the increased mutual repulsion forces in the polypeptide chains, as well as the increased participation of cysteine in thiol-disulphide exchange reactions, may improve protein solubility at an alkaline pH [30]. The latter is potentially supported by the results of amino acid analysis in the present study, showing that cystine, which was absent in the raw material, formed approximately 11–13% of the total amino acids in the hydrolysates (Table 3). It was hypothesized that ultrasonication of the enzymatic/alkaline extracts, either by ultrasonic bath or ultrasound probe, would further improve protein solubility by fueling intermolecular interactions that bring about the conformational changes in the secondary structure of peptides exposing hydrophilic regions to water [32]; however, no significant difference was seen here in the protein recovered in the hydrolysates obtained with or without the ultrasonication of the extracts (*p* > 0.05). This could be accounted for by the overshadowing effect of pH, which had already solubilized the highest possible quantity of protein and peptides before ultrasonication.

### 3.2. Total Phenolic Compounds

Several studies have emphasized the efficiency of ultrasound-assisted extraction in achieving a substantial quantity of phenolic compounds [33]. However, this may be irrelevant because an ultrasound was applied to the extracts after the biomass was sieved out in this study. Therefore, it is unlikely that significantly higher TPC in the ultrasonicated extracts results from the effect of the ultrasound on the disintegration of seaweed cell walls since intact cells are less likely to be present in the extracts after solid residue removal through sieving. One explanation for this observation in the present study is that, although intact cells may not exist after removing the biomass, there might still be residual cellular structures or compounds trapped in cellular remnants soluble at an alkaline pH. Therefore, by generating mechanical forces, ultrasound treatment can further break down these remnants and enhance the release of phenolic compounds. This additional disruption may lead to the elevated recovery of phenolic compounds and justify the observed significant differences between control and ultrasonicated extracts. Another explanation could be the fact that ultrasonication could have yielded smaller peptides and free amino acids, thereby reducing the interactions of protein and algal polyphenols that would otherwise have led to the formation of polyphenol–protein complexes through hydrogen bonding, π-bonding, hydrophobic interactions, and ionic and covalent linkage [34]. Moreover, the Folin–Ciocalteu reagent not only reacts with phenolic compounds but could also react with amino acids containing free hydroxyl groups such as serine, threonine, tyrosine, and glutamic acid. Therefore, a third explanation could be that these amino acids became more accessible for reaction with the Folin–Ciocalteu reagent after the ultrasonication treatment.

Moreover, the observed significant decrease in TPC after ultrasonication through an ultrasonic bath for a longer duration (not seen in extracts treated with an ultrasound probe) might be rooted in the differences in the distribution of ultrasonic energy through bath and probe. In an ultrasonic bath, the ultrasonic energy is unevenly spread, which leads to an uncontrolled and less localized distribution of the sonication effect compared to ultrasound probes [23]. Therefore, upon extended exposure to the extract in an ultrasonic bath, sensitive phenolic compounds might be exposed to degradation. However, more localized ultrasonic energy distribution using ultrasound probes could minimize the risk of the degradation of susceptible phenolic compounds, even if longer ultrasonication times are considered.

### 3.3. Amino Acid Composition

Most of the amino acids were present in the raw material and hydrolysate, except for tryptophan and cysteine (destroyed and converted to cystine, respectively, during acid hydrolysis), as well as hydroxyproline, which is consistent with the results of a study on enzyme-assisted protein extraction from *P. palmata* [11]. Furthermore, histidine was detected in the freeze-dried seaweed, while it was absent in the hydrolysates, which could be related to the imidazole ring of this amino acid. The imidazole ring of histidine is known to be the only amino acid side chain in proteins to act as a pH buffer, where two nitrogen molecules of the ring can protonate or deprotonate to generate the acid or base forms [35]. The change in the protonation state of histidine could bring about alterations in histidine structure and even degradation, which might contribute to its disappearance in the hydrolysates. In addition, cystine was found to be significantly higher in hydrolysates than in free-dried seaweed, likely due to using NAC as a reducing agent during the alkaline extraction stage [11].

In terms of the influence of ultrasonication through bath or probe on amino acid composition, it was witnessed that prolonged exposure of extracts to ultrasonic energy in the bath caused the reduction of all amino acids in hydrolysates (Table 3). However, there were fluctuations in the amount of each amino acid when comparing the hydrolysates obtained after 10 and 30 min of ultrasonication by the probe, which is in line with the results of TPC (see Section 2.2). The uneven distribution of ultrasound energy in the bath might have led to the partial degradation of amino acids in the hydrolysate. In addition, the ratio of essential to non-essential amino acids considerably increased from almost 35% in the raw material to approximately 42% in hydrolysates (Table 3), which indicates the role of enzymatic/alkaline treatment on the increased quantity of essential amino acids in the resulting extracts. The improved ratio of essential to non-essential amino acids in EAEP was also observed by Mæhre et al. [36]. It should be noted that, although prolonged ultrasonication, whether by bath or probe, led to the decreased quantity of amino acids in hydrolysates, it did not cause a considerable change in the ratio of essential to non-essential amino acids in hydrolysates.

### 3.4. In Vitro Antioxidant Properties

The DPPH-scavenging activity of EAEP was attributed to its content of phenolic compounds and low molecular weight peptides and amino acids [34]. However, the significant differences observed in the present study do not correspond with the TPC content of the extracts. Furthermore, ultrasonication using a probe is supposed to exert more localized sonication energy on the extracts, which, in theory, should lead to the formation of peptides with lower molecular weight. Therefore, other attributes could contribute to significantly higher DPPH activity of the control and ULS-B-10 compared with other extracts. One explanation could be that the decline in cystine content in the extracts corresponded to dipped DPPH radical scavenging activity. As cystine levels dropped, the IC_50_ values soared, indicating a probable direct correlation between the cystine content of the hydrolysates and their effectiveness in scavenging DPPH radicals. Accordingly, it was stated that phenolic compounds would have a synergistic effect with S-allyl-l-cysteine, a cysteine derivative, and exhibit strong DPPH radical scavenging activity [37]. Cysteine is a sulfur-containing amino acid characterized by its thiol group. It is known to have biological activity in terms of its antioxidant properties. At the same time, cystine is the dimeric form of cysteine, resulting from the oxidation of two cysteine molecules, and it may exhibit similar properties to cysteine [38]. Nevertheless, this justification seems too good to be true, given that the differences in cystine content among the hydrolysates are insignificant (*p* > 0.05). Another explanation is the possibility of the degradation of bioactive compounds during ultrasonication using a probe due to the elevated degassing effect as well as changes in the characteristics of a medium because of the heating caused by the ultrasonic probe [23]. As to the present study, the removal of gases from the extracts might have caused cavitation bubbles, which generated intense localized energy and mechanical stress that might have degraded susceptible bioactive compounds in the extracts and led to noticeably lower DPPH radical scavenging activity.

Metal ion chelating activity could be attributed to peptides and amino acids in any given extract, with some studies stating that low molecular weight peptides are responsible for Fe^2+^ chelating activity [39]. In contrast, other studies have mentioned that high molecular weight peptides are more effective iron chelators [34]. It was reported that phenolic compounds in *P. palmata* extracts are not effective metal chelators [34], which contrasts with the results obtained in the present study. The Fe^2+^ chelating activity of the extracts here corresponds to the TPC concentrations (Figure 1). TPC content in the sample ultrasonicated in the bath for 10 min is significantly higher than that in the control (*p* < 0.05), which is directly correlated with the significantly lower IC_50_ value of this sample compared with that of the control. Moreover, as stated above, the synergy between phenolic compounds and cysteine derivatives may contribute to higher DPPH radical scavenging activity. A similar synergy would also account for higher metal chelation; however, the possibility of such a synergistic effect in terms of metal chelation remains to be elucidated. In addition, this interpretation should be taken with care because, for compounds to be considered efficient metal chelators, they need to possess functional groups such as hydroxyl (-OH), thiol (-SH), carboxyl (-COOH), phosphoric acid (-PO_3_H_2_), carbonyl (C=O), amino (-NR_2_), sulfide (-S-), or ether (-O-). In contrast, most phenolic compounds only contain a hydroxyl group [39]. The discrepancy in the literature on polyphenol’s role in the metal-chelating activity of seaweed extracts was also reported elsewhere [17]. Concerning the present study, the better chelating activity of ultrasonicated extracts could alternatively be due to the formation of shorter peptides with lower molecular weights or the release of other compounds, such as phospholipids (which are mentioned as suitable metal chelators [40]) from cell remnants after ultrasonication.

From the comparative overview of IC_50_ values for the in vitro antioxidant activities of EAEP in this study and ethanol, water, chloroform, ethyl acetate, and methanol extracts from *P. palmata* reported in other studies, it can be seen that IC_50_ values for DPPH radical scavenging in the present study for all extracts are substantially lower than those for ethanol and water extracts [17] and for chloroform, ethyl acetate, and methanol extracts [24]. However, IC_50_ values for Fe^2+^ chelating activity in ethanol and chloroform extracts were lower than EAEP. Considering the enzymatic treatment performed here using polysaccharidase and protease and the possibility of the formation of amino acids and peptides with low molecular weight, it seems affordable to conclude that peptides in seaweed (or at least in *P. palmata*) extracts are to be cherished for their radical scavenging role. However, the effect of other compounds, such as phospholipids, polysaccharides, and/or polyphenols, on chelate metal ions outweighs that of proteins, peptides, and amino acids in different seaweed extracts.

## 4. Materials and Methods

### 4.1. Seaweed Biomass Preparation

Air-dried *P. palmata* obtained from a batch harvested between late spring and early autumn from Faroe Islands coasts was purchased from a Danish company (DanskTANG, Nykøbing Sj., Denmark). To decide on the feasibility of freeze-drying the biomass before extraction, the dry matter of the retained biomass was calculated after vaporization at 102–105 °C for 24 h and the dry matter content was expressed as (weight) % of the biomass weight. Since the dry matter of the seaweed biomass was 88.2652 ± 0.0075%, the biomass was freeze-dried using a ScanVac CoolSafe freeze-dryer (LaboGene A/S, Allerod, Denmark) to remove as much moisture as possible. The freeze-dried seaweed biomass was then pulverized using a laboratory mill (KnifeTecTM 1095, Foss Tecator, Hillerød, Denmark). Afterwards, the resulting coarse powder was sieved to obtain finer powder and stored in zip-lock plastic bags at −20 °C.

### 4.2. Enzymes and Chemicals

Celluclast^®^ 1.5 L and Alcalase^®^ 2.4 L FG were kindly provided by Novozymes A/S (Bagsværd, Denmark). All the solvents used were of high-performance, liquid chromatography (HPLC) grade and purchased from Lab-Scan (Dublin, Ireland). Amino acid standards were purchased from Sigma-Aldrich (St. Louis, IL, USA). HPLC-grade water was prepared at DTU Food using a Milli-Q^®^ Advantage A10 water deionizing system from the Millipore Corporation (Billerica, MA, USA). BHT, EDTA, and DPPH were obtained from Sigma-Aldrich (Steinheim, Germany). All other chemicals, such as NaOH and NAC, were obtained from Merck (Darmstadt, Germany).

### 4.3. Preparation of EAEP

The enzymatic/alkaline extraction from *P. palmata* was carried out according to Naseri et al. [11] with some modifications to ensure achieving the highest possible solubility of resulting proteins, peptides, and amino acids in liquid extracts. Ten Erlenmeyer flasks (treatments in duplicate to have two batches for each treatment) containing 4 g of biomass powder and 80 mL of deionized water (1:20 *w*/*v*) were placed in a water bath at 50 °C for 1 h for biomass rehydration. Afterward, the pH was adjusted to 8 (recommended in [8] as the most favorable pH for the simultaneous action of enzymes used) using either hydrochloric acid (HCl) or sodium carbonate (Na_2_CO_3_). Celluclast^®^ and Alcalase^®^ were each introduced simultaneously at a concentration of 0.2% of biomass weight, and enzymatic extraction was performed in a water bath at 50 °C for 14 h. Then, the content of each flask was filtered through a sieve (ca. 1 mm mesh size), and the liquid extract was poured into a separate blue-capped bottle and stored at 4 °C. The remaining solid fraction from each flask was re-suspended in 80 mL of the alkaline solution containing 1 g·L^−1^ of NAC, and 4 g·L^−1^ of NaOH, and the first round of alkaline extraction was performed on an orbital shaker at 130 rpm and room temperature for 1.5 h. Two more rounds of alkaline extraction were performed using the solid residue from the previous round suspended in a fresh alkaline solution. The liquid extracts from all three rounds of alkaline extraction were pooled together with enzymatic extract in each blue-capped bottle and stored at 4 °C overnight. After enzymatic/alkaline extraction, the solid residues were dried in an oven at 50 °C and stored at −20 °C before protein content analysis. Next, the pH of EAEP was adjusted to 8.5–9 to ensure the solubility of proteins, peptides, and amino acids. This pH range was found to be efficient in solubilizing the protein, peptide, and amino acid content of the extracts because centrifuging (at 4400× *g* for 15 min at 4 °C) at the end of the process yielded almost no solid residues. Ultrasonication was performed after pH adjustment using either an ultrasonic bath (Cole-Parmer 8893, 42 KHz, Chicago, IL, USA) or ultrasound probe (Qsonica XL2000, 22.5 KHz, Newtown, CT, USA), and the control sample was kept at room temperature when other samples were being ultrasonicated. The treatments were as follows:

- Control: EAEP without ultrasonication
- ULS-B-10: EAEP ultrasonicated in the bath for 10 min.
- ULS-B-30: EAEP ultrasonicated in the bath for 30 min.
- ULS-P-10: EAEP ultrasonicated using the probe for 10 min.
- ULS-P-30: EAEP ultrasonicated using the probe for 30 min.

Then, the extracts were pre-frozen at −20 °C for 2 h and then transferred to a −80 °C freezer for 24 h before they were freeze-dried (LaboGene A/S, Allerod, Denmark). The resulting powders were transferred to zip-lock plastic bags and stored at −80 °C until analysis. It should be noted that all fractions were weighed using a laboratory balance with the readability of 0.01 g at different steps to perform the mass balance calculations.

### 4.4. Protein Content and Recovery

To measure the protein content of the biomass powder, freeze-dried extracts, and oven-dried solid residues collected after enzymatic/alkaline extraction, the total nitrogen content of the samples was determined through the DUMAS combustion method using a fully automated rapid MAX N (Elementar Analysensysteme GmbH, Langenselbold, Germany). Approximately 200 mg of samples were fed into the system, and the exact weight was recorded. The protein content was determined by multiplying the nitrogen content by a factor of 5.0 [11].

Protein recovery in the extracts and solid residues was calculated based on the following equation:

$$\mathrm{Protein}\;\mathrm{recovery}\;\mathrm{in}\;\mathrm{fraction}\;(\%)=\frac{M_{F}\times P_{F}}{M_{S}\times P_{S}}$$

where *M_F_*, *P_F_*, *M_S_*, and *P_S_* stand for the mass of the fraction (extract or solid residue), the protein percentage of the fraction, the mass of the seaweed, and the protein percentage of the seaweed, respectively.

### 4.5. Total Phenolic Content

TPC in the extracts was determined according to [41]. An aliquot (100 μL) of an extract was mixed with 0.75 mL of Folin–Ciocalteu reagent (1:10 diluted) and left at room temperature for 5 min. Sodium bicarbonate (6%, 0.75 mL) was added to the mixture and incubated at room temperature for 90 min. The absorbance was measured at 725 nm using a spectrophotometer (Shimadzu UV mini 1240, Duisburg, Germany). A standard curve was plotted using different concentrations of gallic acid, and the total amount of phenolics was calculated as gallic acid equivalents in µg·mL^−1^.

### 4.6. Amino Acid Profile

Approximately 30 mg of the dried sample was hydrolyzed with 6 M HCl at 110 °C for 18 h. Afterward, the hydrolysates were filtered into 4 mL vials through 0.22 µm cellulose acetate spray filters using 1 mL syringes, and then, 100 µL of the filtered hydrolysates were pipetted into 4 mL vials. The pH adjustment was carried out by slowly adding 1.5 mL of 0.2 M KOH to the hydrolysates, followed by an additional 1.6 mL of ammonium acetate buffer (100 mM; pH 3.1 adjusted with formic acid) to obtain a dilution factor of 32. The amino acid composition was determined by liquid chromatography using mass spectrometry (Agilent 1260 Infinity II Series, LC/MSD Trap, Agilent technologies, Santa Clara, CA, USA) with a BioZen 2.6 µm Glycan, 100 × 2.1 mm (00D-4773-AN) column (Phenomenex, Torrance, CA, USA) connected to a Quadrupole 6120 MS (Agilent Technologies, Santa Clara, CA, USA) with an ESI ion source. The following settings were used: a flow rate of 0.5 mL·min^−1^, a column temperature of 40 °C, 1 µL injection volume, and 16 min run time. A gradient mix of two mobile phases, A (10 mM ammonium formate in acetonitrile) and B (10 mM ammonium formate in MilliQ water) was used as follows: 0–2 min 0–5% phase B, 2–7 min 5–20% phase B, 7–8 min 20–80% phase B, 12.1 min 0% phase B and 12.1–16 min 0% phase B. A mix of amino acid standards containing 17 amino acids (not containing glutamine, tryptophan, or asparagine) was run in five different concentrations to create standard curves. Samples were analyzed, and amino acids were quantitated using MassHunter Quantitative Analysis version 7.0 software. Due to the initial hydrolyzation of the samples, the method cannot detect glutamine, asparagine, tryptophan, or cysteine. Glutamine is hydrolyzed into glutamic acid, while asparagine is hydrolyzed into aspartic acid. Tryptophan and cysteine are destroyed during hydrolysis.

### 4.7. Nitrogen-to-Protein Conversion Factor

Nitrogen-to-protein conversion factors were calculated based on the protein content obtained in Section 4.4 (considering the nitrogen-to-protein factor of 5) and the protein content (%) based on total amino acids achieved from Section 4.6 as follows:

$$\mathrm{Nitrogen-to-protein}\;\mathrm{conversion}\;\mathrm{factor}=\frac{5\times P_{aa}}{P_{D}}$$

where *P_aa_* and *P_D_* denote protein content (%) based on total amino acids and protein content (%) based on the protein content (%) obtained from DUMAS by considering the conversion fact of 5 [11].

### 4.8. DPPH Radical Scavenging Activity

DPPH radical scavenging activity was measured according to [42], modified using microtiter plates and a multiplate reader. The extracts were dissolved in distilled water to acquire solutions with different concentrations. Afterward, 150 μL of the solution were mixed with 150 μL of the 0.1 mM ethanolic solution of DPPH and then kept in the dark at ambient temperature for 30 min. The absorbance was read at 515 nm by an Eon™ microplate spectrophotometer (BioTek Instruments, Inc., Winooski, VT, USA). For the blank, distilled water was used instead of the sample. Control was prepared with 150 μL of the sample and 150 μL of 95% ethanol. All the measurements were carried out in triplicate. For positive control, a BHT solution (0.2 mg·mL^−1^) was used. DPPH-scavenging capacity was derived as follows:

$$\mathrm{DPPH}\;\mathrm{scavenging}\;\mathrm{activity}\;(\%)=\left(1-\frac{\left(A_{s}-A_{c}\right)}{A_{b}}\right)\times 100$$

where *A_s_*, *A_c_*, and *A_b_* stand for the absorbance of the sample, control, and blank, respectively. Furthermore, sample concentrations (mg protein·mL^−1^) that needed to inhibit 50% of DPPH activity (IC_50_ values) were determined by drawing dose–response curves.

### 4.9. Fe^2+^ Chelating Activity

The Fe^2+^ chelating activity of the extracts was measured according to [43] modified using microtiter plates and a multiplate reader. The extracts were dissolved in distilled water to obtain different concentrations. Then, each extract solution (200 μL) was blended with distilled water (270 μL) plus ferrous chloride 2 mM (10 μL). The reaction was blocked after 3 min using 20 μL of ferrozine solution 5 mM. The mixture was then shaken vigorously. After 10 min at ambient temperature, the absorbance was read at 562 nm by an Eon™ microplate spectrophotometer (BioTek Instruments, Inc., Winooski, VT, USA). For the blank, distilled water was used instead of the sample. Sample control was prepared without adding ferrozine. All the measurements were carried out in triplicate. For positive control, 0.06 mM EDTA was used. The metal-chelating activity was calculated as follows:

$${\mathrm{Fe}}^{2+}\;\mathrm{chelating}\;\mathrm{activity}\;(\%)=\left(1-\frac{\left(A_{s}-A_{c}\right)}{A_{b}}\right)\times 100$$

where *A_s_*, *A_c_*, and *A_b_* stand for the absorbance of the sample, control, and blank, respectively. Also, sample concentrations (mg protein·mL^−1^) that needed to chelate 50% of Fe^2+^ (IC_50_ values) were determined by drawing dose–response curves.

### 4.10. Statistical Analysis

The acquired data were analyzed via Analysis of Variance (ANOVA), and differences between means were determined using the Tukey test. All the statistical operations were performed in OriginPro 2023 (OriginLab Co., Northampton, MA, USA). Differences were considered significant at *p* < 0.05.

## 5. Conclusions

In conclusion, the previously used nitrogen-to-protein conversion factor of 5 was too high for the extracts, as evidenced by the results obtained regarding the total amino acid compositions as a measure of protein content by accounting for the water gained during the protein hydrolysis stage. Enzymatic/alkaline extraction in this study facilitated the development of antioxidant extracts from *P. palmata*. All the extracts generally exhibited favorable radical scavenging and metal-chelating activities. Ultrasonication, either by bath or probe, seemed to have no significant effect on the degree of hydrolysis and protein recovery in the extracts. According to the results of the study, it is advisable to exert ultrasonication for a short time (e.g., 10 min) by using an ultrasonic bath on the extracts after removing the biomass because the resulting extract showed significantly higher metal-chelating activity than the control sample. Consistently, the extract ultrasonicated in the bath for a shorter time was the only sample with no significant difference from the control sample in terms of their potency to scavenge free radicals. To top it off, the extract ultrasonicated in the bath for the shorter period contained a significantly higher content of phenolic compounds compared with the one sonicated the same way but for a longer period and with the extracts treated with post-extraction ultrasonication using the ultrasound probe. However, the mutual relationships and interactions between phenolic compounds, peptides and amino acids, and other compounds such as carbohydrates, should be considered to make more accurate inferences. Overall, according to the results obtained from the present study, treating EAEP with ultrasound energy in an ultrasonic bath for a short time could be a new and economical way to improve their bioactivity. Future research endeavors should investigate post-extraction ultrasonication’s effects on the various properties of extracts obtained from different seaweed and microalgae species. Additionally, there is ample opportunity to explore the efficiency of different extraction techniques, whether used individually or in combination, based on the results observed following post-extraction treatment using various ultrasonic methods. Furthermore, it is advisable for upcoming studies to analyze the constituents of resulting extracts, specifically examining peptides, phenolic compounds, and polysaccharides. Such analysis will provide a clearer understanding of the compounds responsible for the observed properties.

## Figures and Tables

**Table 1:** Protein content (% dry matter), degree of hydrolysis (DH), and protein recovered in liquid and solid fractions after enzymatic/alkaline extraction from *P. palmata* (PRE and PRSR).

	Dried Seaweed	Control	ULS-B-10	ULS-B-30	ULS-P-10	ULS-P-30
Protein (%)	12.85 ± 0.63	11.20 ± 0.16	10.96 ± 0.20	10.25 ± 0.66	10.75 ± 0.15	10.67 ± 0.34
DH (%)	-	28.88 ± 1.34	33.19 ± 4.86	29.43 ± 0.15	25.39 ± 2.41	26.86 ± 3.95
PRE * (%)	-	93.00 ± 0.59	93.60 ± 0.66	93.91 ± 0.52	93.83 ± 1.50	91.39 ± 0.17
PRSR ** (%)	-	3.30 ± 1.65	3.99 ± 0.69	4.17 ± 1.20	4.65 ± 0.00	3.97 ± 0.22

Data were expressed as mean ± standard deviation (*n* = 2). No significant difference was observed among the samples in terms of protein content, DH, PRE, and PRSR (*p* > 0.05). Control: no ultrasonication; ULS-B-10: ultrasonication by bath for 10 min; ULS-B-30: ultrasonication by bath for 30 min; ULS-P-10: ultrasonication by probe for 10 min; ULS-P-30: ultrasonication by probe for 30 min. * Protein recovered in enzymatic/alkaline extracts. ** Protein remaining in solid residue after enzymatic/alkaline treatment.

**Table 2:** Protein content of freeze-dried *P. palmata* and its hydrolysates based on different nitrogen-to-protein conversion factors (NCF) and the calculation of the conversion factors for each sample.

	Protein (%): NCF 6.25	Protein (%): NCF 5	Protein (%): NCF 4	Protein (%) Based on TAA *	NCF Based on TAA	Protein (%) Based on TAAPC **	NCF Based on TAAPC
Dried seaweed	16.05 ± 0.55 ^a^	12.85 ± 0.63 ^a^	10.27 ± 0.35 ^a^	12.34	4.80	11.68	4.54
Control	14.00 ± 0.14 ^ab^	11.20 ± 0.16 ^ab^	8.96 ± 0.09 ^ab^	10.31	4.60	8.94	3.99
ULS-B-10	13.70 ± 0.18 ^b^	10.96 ± 0.20 ^b^	8.76 ± 0.11 ^b^	10.25	4.67	8.72	3.97
ULS-B-30	12.80 ± 0.59 ^b^	10.25 ± 0.66 ^b^	8.19 ± 0.37 ^b^	9.67	4.71	8.23	4.01
ULS-P-10	13.44 ± 0.13 ^b^	10.75 ± 0.15 ^b^	8.60 ± 0.08 ^b^	10.13	4.71	8.41	3.91
ULS-P-30	13.33 ± 0.30 ^b^	10.67 ± 0.34 ^b^	8.53 ± 0.19 ^b^	9.82	4.60	8.97	4.20

Data were expressed as mean ± standard deviation (*n* = 2). The letters ^a^ and ^b^ denote significant differences among the treatments (*p* < 0.05). Control: no ultrasonication; ULS-B-10: ultrasonication by bath for 10 min; ULS-B-30: ultrasonication by bath for 30 min; ULS-P-10: ultrasonication by probe for 10 min; ULS-P-30: ultrasonication by probe for 30 min. * Total amino acid. ** Total amino acid as a measure of protein content (by considering water gained during protein hydrolysis).

**Table 3:** Amino acid (mg·g^−1^ sample) contents of dried seaweed and EAEP.

	Dried Seaweed	Control	ULS-B-10	ULS-B-30	ULS-P-10	ULS-P-30
PHE *	4.61 ± 0.10	2.99 ± 0.18	3.01 ± 0.07	2.79 ± 0.19	3.02 ± 0.09	2.87 ± 0.16
LEU *	8.60 ± 0.25	5.71 ± 0.41	5.86 ± 0.15	5.43 ± 0.34	5.73 ± 0.03	5.70 ± 0.54
ILE *	3.96 ± 1.48	3.65 ± 0.16	3.59 ± 0.03	3.39 ± 0.25	3.57 ± 0.10	3.54 ± 0.32
MET *	2.04 ± 0.10	1.61 ± 0.02	1.66 ± 0.01	1.58 ± 0.13	1.58 ± 0.08	1.59 ± 0.11
TYR *	4.05 ± 0.25	2.75 ± 0.25	2.79 ± 0.05	2.65 ± 0.33	2.71 ± 0.22	2.67 ± 0.11
PRO	7.19 ± 0.16	5.26 ± 0.44	5.20 ± 0.20	4.99 ± 0.41	5.21 ± 0.10	5.25 ± 0.41
VAL *	8.60 ± 0.21	6.57 ± 0.57	6.30 ± 0.49	6.04 ± 0.53	6.19 ± 0.24	6.39 ± 0.90
ALA	11.17 ± 0.34	8.11 ± 0.01	8.25 ± 0.34	7.54 ± 0.10	8.25 ± 0.12	8.14 ± 1.25
THR *	5.47 ± 0.12	4.22 ± 0.56	3.70 ± 0.27	3.59 ± 0.01	3.81 ± 0.08	4.00 ± 0.14
GLY	8.80 ± 0.22	6.28 ± 0.87	6.10 ± 0.15	6.04 ± 0.10	6.40 ± 0.56	6.48 ± 0.78
SER	8.08 ± 0.12	6.09 ± 0.30	6.33 ± 0.09	5.47 ± 0.02	6.31 ± 0.03	5.63 ± 0.52
ARG	6.83 ± 0.19	3.87 ± 0.41	3.75 ± 0.17	3.65 ± 0.01	3.63 ± 0.19	3.72 ± 0.82
HIS *	1.22 ± 0.06	ND **	ND	ND	ND	ND
LYS *	4.37 ± 0.19	3.36 ± 0.19	3.52 ± 0.21	3.24 ± 0.05	3.28 ± 0.04	3.20 ± 0.05
GLU	19.46 ± 0.46	15.39 ± 0.08	15.19 ± 0.83	14.41 ± 0.67	15.47 ± 0.52	14.18 ± 1.17
C-C *	0.51 ± 0.16	13.17 ± 1.06	13.19 ± 1.22	12.49 ± 0.80	11.99 ± 0.98	11.75 ± 0.09
ASP	18.44 ± 0.57	14.07 ± 0.01	14.09 ± 0.30	13.42 ± 0.02	14.16 ± 0.09	13.09 ± 0.76
TAA ***	123.4	103.1	102.53	96.72	101.31	98.2
EAA	43.43	44.03	43.62	41.2	41.88	41.71
EAA/TAA	0.351	0.427	0.425	0.425	0.413	0.424

Data were expressed as mean ± standard deviation (*n* = 2). No significant difference was observed among the samples in terms of amino acids (*p* > 0.05). Control: no ultrasonication; ULS-B-10: ultrasonication by bath for 10 min; ULS-B-30: ultrasonication by bath for 30 min; ULS-P-10: ultrasonication by probe for 10 min; ULS-P-30: ultrasonication by probe for 30 min. * Essential amino acids (EAA) in human nutrition [8]. ** Not detected. *** Total amino acids.

**Table 4:** Comparison of IC_50_ values for in vitro antioxidant activities of EAEP with other types of extracts in different studies from *P. palmata*.

	EAEP					Ethanol Extract [1]	Water Extract [1]	Chloroform Extract [2]	Ethyl Acetate Extract [2]	Methanol Extract [2]
	Control	ULS-B-10	ULS-B-30	ULS-P-10	ULS-P-30					
DPPH *	0.07	0.09	0.14	0.17	0.21	1.16	0.57	>1	0.78	>1
Fe^2+^ Ch **	1.40	1.12	1.17	1.24	1.21	0.84	0.75	NR ***	NR	NR

Data were expressed as mean ± standard deviation (*n* = 2). Control: no ultrasonication; ULS-B-10: ultrasonication by bath for 10 min; ULS-B-30: ultrasonication by bath for 30 min; ULS-P-10: ultrasonication by probe for 10 min; ULS-P-30: ultrasonication by probe for 30 min. * IC_50_ values (mg·mL^−1^) of different extracts for DPPH radical scavenging activity. ** IC_50_ values (mg·mL^−1^) of different extracts for Fe^2+^ chelating activity. *** Not reported.

## Data Availability

The data presented in this study are available in the article.
